# Endothelial Cell: Lactate Metabolic Player in Organ Regeneration

**DOI:** 10.3389/fcell.2021.701672

**Published:** 2021-08-18

**Authors:** Lanlan Zhang, Xuezhen Gui, Xin Zhang, Yujing Dai, Xiangjun Wang, Xia Tong, Shasha Li

**Affiliations:** ^1^State Key Laboratory of Biotherapy of China, Division of Pulmonary Diseases, West China Hospital of Sichuan University, Chengdu, China; ^2^Department of Gastroenterology, West China Hospital of Sichuan University, Chengdu, China; ^3^First Affiliated Hospital of Sun Yat-sen University, Guangzhou, China; ^4^Athinoula A. Martinos Center for Biomedical Imaging, Department of Radiology, Massachusetts General Hospital, Harvard Medical School, Boston, MA, United States

**Keywords:** endothelial cell, metabolism, regeneration, angiogenesis, lactate

## Introduction

Endothelial cells (ECs), as the “gatekeeper” by controlling the extravasation of circulating cells into tissues, play an essential role in regulating tissue homeostasis (Eelen et al., 2018). By altering their production of cytokines, chemokines, and adhesion molecules, endothelial cells control the traffic of immune cells into injury sites (Gerhardt and Ley, 2015). Injury or dysfunction of ECs is known to contribute to many pathologies, including metabolism-related disease, in which loss of the glycolytic activator phosphofructokinase-2/fructose-2,6-bisphosphatase isoform 3 in ECs impairs vessel formation (Li et al., 2019). Besides, ECs also contribute to homeostasis and regeneration *via* “angiocrine” signaling (Ding et al., 2014; Cao et al., 2016; Rafii et al., 2016), which secretes “angiocrine” cytokines for tissue regeneration.

Lactate (2-hydroxypropanoic acid) is divided into two types, L-lactate and D-lactate. The former is the focus of this review; L-lactate means lactate. Lactate is stored in the body and is the terminal product of anaerobic metabolism, considered a metabolic waste. In recent years, it has been discovered that lactate is not simply waste but fertilizer. However, EC's function on lactate metabolism for peripheral tissue regeneration remains to be fully elucidated.

This paper focuses on the role of endothelial–lactate interactions in regulating metabolic microenvironment on tissue regeneration and propose that endothelial–lactate interactions affect the microenvironment through two pathways except for the classical angiogenesis pathway; (1) lactate uses endothelium as a gatekeeper to regulate the microenvironment through immune cells, affecting the regeneration of the damaged tissues; (2) ECs secrete lactate to repair the organ regeneration caused by the damaged tissues.

## Endothelial Cells “Gatekeeper” Function for Tissue Regeneration

The role of “gatekeeper” is a vital role for ECs. ECs play an essential role in the chemotaxis of neutrophils by inflammation and the recruitment of macrophages for homeostasis. Former studies focused on the classical hypothesis that cancer cells ensure sufficient oxygen and nutrient supply for proliferation through lactate-induced secretion of vascular endothelial growth factor (VEGF), forming the new vessels (form a barrier for “gatekeeper”) (Kumar et al., 2007; Longchamp et al., 2018). Lactate uses the “lactate shuttle” in ECs to shuttle between cells and affect cell differentiation in cancers (de la Cruz-López et al., 2019). Even under quiescent conditions, ECs can use glycolysis to convert glucose into lactate (De Bock et al., 2013). Simultaneously, lactate as a metabolizable anion may lead to Cl^−^ egress from ECs, giving rise to ECs swelling and functional barrier changes (Hoffmann et al., 2009).

In recent years, that lactate promotes organ regeneration through ECs and that the classic lactate enhances ECs angiogenesis have been put on the agenda. ECs and lactate transporter play an essential role in lactate-induced tissue regeneration. The brain is an excellent example of lactate going through ECs for tissue regeneration for the “gatekeeper” role. Exercise can increase lactate production from skeleton muscle and promote lactate penetration through the blood–brain barrier. Morland et al. (2017) reported that high-intensity exercise induces the release of lactate from the muscle strains through the vascular endothelial lactate receptor (hydroxycarboxylic acid receptor 1) receptor. Previous studies have also suggested that cerebral vascular ECs highly express monocarboxylate transporter (MCT), a lactate transporter, allowing lactate to shuttle through the ECs' cell membrane, which works as a path for the lactate-induced metabolism of the entire brain (Zhang et al., 2014). Lactate in the blood can be transferred into ECs through MCT1 and then transported to the brain. After lipopolysaccharide stimulation, MCT-1 of the ECs decreased, and interleukin 1β accumulates in the brain, followed by a rise in lactate content (Boitsova et al., 2018). Lev-Vachnish et al. (2019) blocked the expression of MCT2 in ECs *in vivo*, which transports lactate to the brain, and finally found that mouse brain neuron regeneration was enhanced. Therefore, the lactate transporter plays a “gatekeeper” role in transporting lactate through the endothelial barrier to the damaged organ.

The mechanism of how the “gatekeeper” function of ECs on lactate affects the brain's cognitive function through supporting tissue regeneration is also worth discussing. Wang et al. (2019) illustrated how the brain ECs maintained lactate homeostasis and contributed to adult hippocampal neurogenesis and cognitive functions. Conditional phosphatase and tensin homolog (PTEN) knockout in ECs increased the outflow of lactate from the blood through ECs to nearby tissue, and administering lactate to wild-type animals impaired adult hippocampal neurogenesis. Thus, the brain lactate was upregulated, suggesting that ECs can maintain the lactate-induced metabolic homeostasis. Simultaneously, the expression of MCT1 decreased with the knockout of PTEN in ECs, and neurocognitive function also declined in the hippocampus of mice, indicating that PTEN of ECs regulates the metabolism of lactate in the brain by upregulating the expression of MCT1. Moreover, deletion of Akt1 in ECs restored MCT1 expression, decreased lactate levels, and normalized hippocampal neurogenesis and cognitive function in PTEN mutant mice. As a virtual channel of “gatekeeper,” lactate transporter carries lactate in the blood to neurons through critical signal transduction pathways of ECs to improve neurogenesis and cognitive functions.

**Figure 1:**
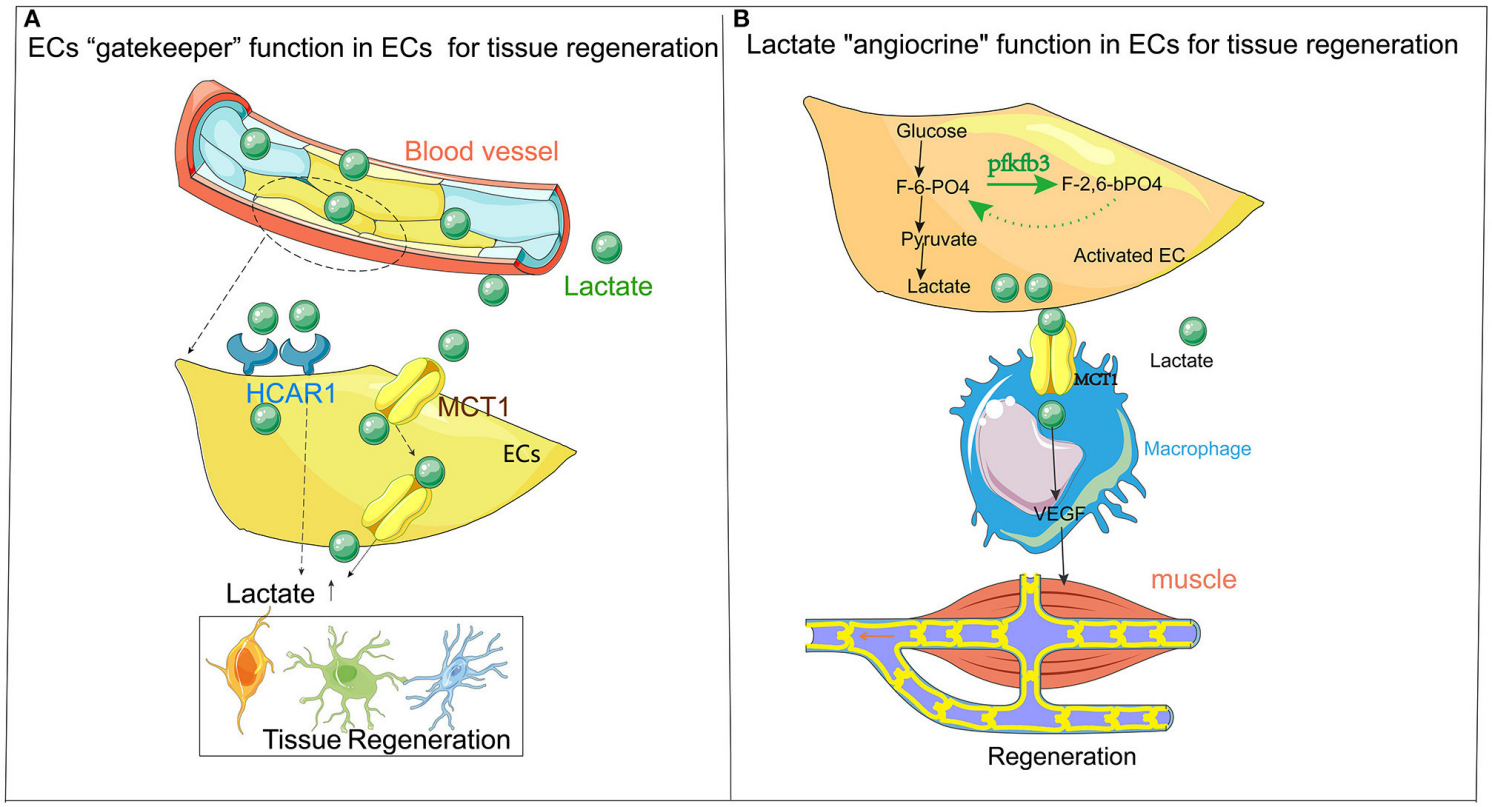
**(A)** ECs are the “gatekeepers” of lactate. Lactate metabolism plays a vital role in the regeneration of tissues (especially in the nervous system). The accumulation of lactate often leads to neurodegenerative diseases. The primary mechanism of action is the “gatekeeper” function of ECs as a metabolite, allowing lactate to be carried through the bloodstream to the site that needs damage and repair with the transporter (HCAR1 and MCT1) on ECs, thereby affect the damage and repair of tissues. **(B)** Flow chart describing “classical” ECs release lactate for local metabolism and tissue regeneration. The endothelium itself is a high-metabolic organ, causes lactate accumulation through endothelial activation, which affects the regeneration and repair of surrounding tissues. The primary mechanism is that lactate (after exercise) undergoes anaerobic metabolism in ECs and finally changes from glucose to lactate, following an amount of lactate accumulated in ECs. Through ECs' transporter, such as MCT1, the lactate is released from ECs to recruit immune cells such as macrophages. The macrophages release VEGF and other cytokines to the surrounding ischemic tissues (such as muscle tissue), producing regeneration and repair. HCAR1, lactate receptor Vascular endothelial growth factor; MCT1, monocarboxylate transporter 1; ECs, endothelial cell.

Therefore, based on these particular steps of metabolism, ECs can reprogram the lactate and promote the regeneration of the damaged tissue. Finally, lactate is released and transported through the lactate transporter on ECs, passing through the endothelial barrier to the damaged tissues for tissue repair (Figure 1A).

## Lactate “Angiocrine” Function in Endothelial Cells for Tissue Regeneration

ECs act as a “gatekeeper,” controlling lactate secretion for organ-like brain regeneration in previous reports. ECs also can maintain lactate homeostasis and facilitate tissue repair and regeneration *via* “angiocrine” signaling. For instance, Jag1 derived from lung ECs ensures control of the lung regeneration after bleomycin injection (Cao et al., 2016); in the mouse brain, quiescence of neural stem cells can be sustained by neurotrophin-3 released from the brain endothelium (Ottone et al., 2014).

ECs relied on glycolysis rather than on oxidative phosphorylation for adenosine triphosphate production, and the loss of the glycolytic activator phosphofructokinase-2/fructose-2,6-bisphosphatase isoform 3 (PFKFB3) in ECs impaired vessel formation. EC's activation enhanced the glycolytic regulator PFKFB3 when along with glucose, it was oxidized during glycolysis. Deletion of PFKFB3 in ECs prevents revascularization and lactate reprogramming, and the addition of the lactate to PFKFB3-knockdown cells rescued the suppression of EC proliferation and migration (De Bock et al., 2013; Schoors et al., 2014; Xu et al., 2014). Dual inhibition of PFKFB3 and VEGF normalized tumor vasculature, reduced lactate production, and improved chemotherapy in glioblastoma (Zhang et al., 2020b). Moreover, the lactate promoted EC proliferation and migration in PFKFB3-knockdown cells. Eventually, PFKFB3 can control lactate secretion in ECs. However, whether the ECs acted as a “reservoir” for lactate through PFKFB3 is rarely reported.

The function of macrophages to promote fibrosis and regeneration has been well-studied (Cui et al., 2021). However, few studies on ECs regulate macrophages by releasing lactate and macrophages as mediators for tissue regeneration and repair. Zhang et al. (2020a) exploited lactate–EC metabolic communication that contributes to tissue regeneration uniquely. They found that endothelial PFKFB3 is required for ischemia-induced muscle revascularization and regeneration and that knocking PFKFB3 in muscle ECs impairs muscle function upon ischemia. Loss of endothelial PFKFB3 in ECs increased monocyte recruitment during ischemia but dampened M2 macrophage polarization. Muscle ECs use lactate as a signal of sprouting within the muscle microenvironment, promoting M2-like macrophage functional polarization and augmentation VEGF secretion from macrophages. Eventually, macrophages create a positive feedback loop that can stimulate EC's angiogenesis further.

It is novel that ECs metabolically collaborate with macrophages to repair muscle, efficiently sharing the energetic substrate glucose *via* the glycolysis to lactate. Finally, ECs are no longer a simple barrier but are activated by tissue damage and activated ECs, which become a reservoir of metabolites such as lactate. ECs with a large amount of lactate are used as raw material and fuel to combine with the lactic acid transporter of immune cells to promote the release of cytokines, amplifying the repairing effect of immune cells in damaged tissues (Figure 1B).

Traditionally, ECs are the blood barrier in which oxygen, nutrients, and metabolites can be transported and exchanged with the blood flow, among which lactate is a vital metabolite shuttled through the “switch” action of ECs. Lactate passes through the transporter of ECs, allowing the lactate produced by other tissues (such as muscles) to be carried through the blood to the targeted organs (such as neurons), promoting tissue regeneration. However, ECs can also serve as a “reservoir” for the release of lactate, allowing the lactate with “angiocrine” function to be released from ECs, and the damaged tissue can be repaired with lactate. Therefore, the “gatekeeper” role of ECs can allow activated ECs to act as a gated channel to release lactate when repairing other tissues. However, the angiocrine function of ECs is achieved by endothelial activation. Then, lactate is released, and immune cells are recruited, which are undergoing tissue regeneration and repair.

## Conclusion and Future Prospects

Here, in addition to the angiogenesis effect of lactate on ECs, this paper further discusses the “gatekeeper” function effect of ECs on the metabolism of lactate for tissue regeneration and how lactate penetrates ECs to affect the surrounding tissues of blood vessels, such as the formation of brain nerves. More interestingly, we also discuss the ECs as a “reservoir” of lactate, which can secrete lactate from ECs, thus affecting the regeneration of nearby tissues.

In EC microenvironment, lactate contributes metabolic crosslinks between immune cells and stromal cells, acting as a “lactate-shuttle” in the damaged tissue. Lactate is no longer a waste product derived from anaerobic metabolism; instead, it is a powerful molecule tool that contributes to EC's angiocrine function, favoring tissue regeneration. Thus, new tools, including tracing lactate location and consumption and transporting lactate to damaged tissue, deserve further study.

Although several profoundly illustrated mechanisms have been identified as causes of organ regeneration in lactate-related microenvironments, limited evidence supports metabolic cross talk between immune cells or supportive tissues and ECs, which is obviously another challenge for future research in the EC metabolism field. Despite the scarcity of researches related to ECs' molecular function of regulating lactate, translational research on lactate metabolism in ECs has transferred some fundamental aspects of the regeneration-related disease to the clinical aspects. Clinical researchers working in regeneration disease are encouraged to utilize lactate metabolism to treat ischemia or neuron degeneration.

In summary, ECs can promote blood vessel formation in an environment with high lactate levels and act as a gate of lactate, controlling lactate secretion and affecting tissue regeneration. Finally, activated ECs also serve as a storehouse of lactate, and the activated ECs can secrete lactate to promote the regeneration of surrounding tissues.

## Author Contributions

LZ: conception or design of the work and critical revision of the article. XG: drafting the article and data collection. XZ and XT: drafting the manuscript and revision of the article. YD: drafting the article. XW: drafting the manuscript. SL: critical revision of the article.

## Conflict of Interest

The authors declare that the research was conducted in the absence of any commercial or financial relationships that could be construed as a potential conflict of interest.

## Publisher's Note

All claims expressed in this article are solely those of the authors and do not necessarily represent those of their affiliated organizations, or those of the publisher, the editors and the reviewers. Any product that may be evaluated in this article, or claim that may be made by its manufacturer, is not guaranteed or endorsed by the publisher.
